# Development of a model of medication review for use in clinical practice: Bristol medication review model

**DOI:** 10.1186/s12916-021-02136-9

**Published:** 2021-11-10

**Authors:** D. McCahon, R. E. Denholm, A. L. Huntley, S. Dawson, P. Duncan, R. A. Payne

**Affiliations:** Centre for Academic Primary Care, Population Health Sciences, University of Bristol, Canynge Hall, Bristol, BS8 2PS UK

**Keywords:** Medicine optimisation, Prescribing, Medication review

## Abstract

**Background:**

Medication review is a core aspect of medicine optimisation, yet existing models of review vary substantially in structure and content and are not necessarily easy to implement in clinical practice. This study aimed to use evidence from the existing literature to identify key medication review components and use this to inform the design of an improved review model.

**Methods:**

A systematic review was conducted (PROSPERO: CRD42018109788) to identify randomised control trials of stand-alone medication review in adults (18+ years). The review updated that by Huiskes et al. (BMC Fam Pract. 18:5, 2017), using the same search strategy implemented in MEDLINE and Embase. Studies were assessed using the Cochrane risk of bias tool. Key review components were identified, alongside relevant clinical and health service outcomes. A working group (patients, doctors and pharmacists) developed the model through an iterative consensus process (appraisal of documents plus group discussions), working from the systematic review findings, brief evidence summaries for core review components and examples of previous models, to agree on the main purpose of the review model, overarching model structure, review components and supporting material.

**Results:**

We identified 28 unique studies, with moderate bias overall. Consistent medication review components included reconciliation (26 studies), safety assessment (22), suboptimal treatment (19), patient knowledge/preferences (18), adherence (14), over-the-counter therapy (13) and drug monitoring (10). There was limited evidence from studies for improvement in key clinical outcomes. The review structure was underpinned by patient values and preferences, with parallel information gathering and evaluation stages, feeding into the final decision-making and implementation. Most key components identified in the literature were included. The final model was considered to benefit from a patient-centred, holistic approach, which captured both patient-orientated and medication-focused problems, and aligned with traditional consultation methods thus facilitating implementation in practice.

**Conclusions:**

The Bristol Medication Review Model provides a framework for standardised delivery of structured reviews. The model has the potential for use by all healthcare professionals with relevant clinical experience and is designed to offer flexibility of implementation not limited to a particular healthcare setting.

**Supplementary Information:**

The online version contains supplementary material available at 10.1186/s12916-021-02136-9.

## Background

Medicines are a fundamental intervention used by health services to improve health and well-being. In patients with long-standing or several health problems, the use of multiple medicines—polypharmacy—is commonplace [1]. If used appropriately, polypharmacy may lead to improvements in clinical outcomes [2]. However, it may also be associated with a range of adverse consequences including hazardous prescribing [3] and reduced quality of life [4].

The process of medicine optimisation serves to ensure medicines are used in the most effective and safe manner possible [5]. The Royal Pharmaceutical Society describes four guiding principles to achieve this, applicable to both front-line care and service development: understanding the patient’s experience, ensuring evidence-based choice of medicines, ensuring the safety of medicines use and embedding medicine optimisation in routine practice [6]. Medication review forms a critical part of the process. It has been defined by the UK’s National Institute for Health and Care Excellence (NICE) as “a structured, critical examination of a person’s medicines with the objective of reaching an agreement with the person about treatment, optimising the impact of medicines, minimising the number of medicine-related problems and reducing waste” [7]. Other definitions may be more focused; for example, the Pharmaceutical Care Network of Europe considers reviews to “entail detecting drug-related problems and recommending interventions” [8]. The structure and content of reviews also vary depending on the nature of clinical information available and the involvement (or otherwise) of the patient in the review process [8]. In addition, clinical guidance differs in how it specifies the way a review should be carried out: the WHO technical report on medicine safety in polypharmacy describes a seven-step model [9], whereas the NICE medicine optimisation guideline does not outline a particular approach [7].

Some studies have demonstrated medication review to be associated with improvements in certain clinical outcomes in specific patient groups. For example, significant reductions in drug-related problems have been achieved with medication review in people with diabetes [10] and heart failure [11]. In addition, complex interventions, incorporating elements such as clinical informatics and education, have been shown to reduce problematic prescribing [12, 13]. However, a systematic review of the literature found a majority of pharmacist-led interventions in residential aged care facilities, of which medication review was a common component, were ineffective [14]. Another review focusing on older people with multimorbidity and polypharmacy found mixed effects on outcomes [15]. A further systematic review by Huiskes et al. found that an isolated medication review had minimal impact on clinical outcomes and no effect on the quality of life, attributing this in part to the insufficient standardisation of reviews and differing review objectives, and going as far to suggest such reviews should not be part of standard care [16]. Nevertheless, structured review is widely used internationally, despite significant inconsistencies in approach including in both service design and clinical issues addressed [17, 18].

The aim of the current study was to review the existing literature to identify the active elements of a medication review and use the findings to develop an evidence-based, structured model of medication review for use in clinical practice.

## Methods

### Systematic review study design

The protocol for the systematic review has been published on PROSPERO (reference CRD42018109788) [19]. The purpose of the literature review was to establish the structure and components commonly included within medication review strategies. This was done by updating an existing review [16], to which revised inclusion/exclusion criteria were additionally applied.

The starting point was a systematic review published in 2017 by Huiskes et al. [16], the aim of which was to summarise the evidence of medication review as a stand-alone short-term intervention (e.g. as performed in clinical practice), irrespective of the patient population and the outcome measures. Huiskes et al. identified 33 randomised controlled trials (RCTs) published before September 2015 investigating the effectiveness of medication review.

### Data sources and searches

An updated literature search, using the same search strategy as Huiskes et al. for the period 2015 to 2018, was also performed in MEDLINE and Embase databases. Reference lists and citations of included papers were also examined for relevant articles. The search strategy is presented in Additional File 1: Appendix 1. Papers were managed in EndNote.

### Study selection

The study selection criteria were modified slightly from that of Huiskes et al., to increase relevance in terms of informing the development of a new review model. We applied these revised inclusion/exclusion criteria both to the new studies identified and to the previous studies identified in the Huiskes’ review. Consistent with the approach taken by Huiskes, we applied no restrictions to the outcome measures.

All RCTs investigating the effectiveness of medication review (including protocol papers) were considered for inclusion, where the intervention was subjectively judged by the research team to meet the definition of medication review as stated by the NICE guidelines for medicine optimisation [7]. The study design needed to include a cross-sectional intervention delivered within a primary or secondary care setting, by any healthcare professional, with the intervention involving patient participation in the form of provision of information and/or involvement in the discussion and decision-making. The study population was restricted to adults, aged 18 years or over.

Studies were excluded where the medication review (1) targeted a specific disease, condition or a single class of drug; (2) aimed to solely improve patient knowledge and adherence or reduce costs; (3) formed part of a complex intervention or included a co-intervention (e.g. discharge counselling, non-pharmacological intervention, professional education); (4) was conducted within a palliative care setting; or (5) involved no direct patient participation. We also excluded articles in languages other than English.

Reviewer DM ran the database searches. The titles and abstracts were screened by DM and RP/RD independently. Full-text screening was conducted independently by DM and RD. Disagreement between reviewers was resolved through discussion with RP.

### Data extraction and risk of bias assessment

The characteristics of the study (e.g. setting, population, outcomes), medication review components (e.g. clinical areas, question types, review facilitators), underpinning behavioural change theory (which could be used to inform specific approaches to model design) and outcome data were extracted from the full-text articles into a data recording proforma by one reviewer and checked by a second reviewer.

The risk of bias in eligible studies was independently assessed by two of the three reviewers (DM, RD, RP) using the Cochrane risk of bias tool [20], with disagreements resolved through discussion with a third reviewer.

### Analysis

Thematic analysis was used by DM to develop a framework to classify the components of the different medication review strategies. RD piloted the framework in a random selection of 25% of studies. Comparison to identify disagreements was undertaken with discrepancies being discussed with RP to agree and refine the framework and improve face validity. The finalised framework (Additional File 1: Appendix 2) was used independently by DM and RD to code all included studies.

Outcomes were categorised into five main overarching groups, following discussion between DM, RD and RP based on the experience of the topic area: safety, efficacy, service use, patient experience and mortality. The latter two categories were added as it was apparent that the first three categories prespecified by the protocol did not capture the full range of outcomes being reported. *P*-values of < 0.05 were considered statistically significant.

Each intervention component was mapped against corresponding study outcomes, with a view to conducting a meta-regression of the two.

### Stakeholder working group

The medication review model was constructed through an iterative process through consultation via meetings and offline work with a UK-based stakeholder working group comprising clinical (RP) and non-clinical (DM) members of the research team, two GPs, a geriatrician, two clinical pharmacists with roles in medicine optimisation and two patients with experience in patient and public engagement with medicine optimisation research. The group was recruited through existing networks and selected to ensure diversity of membership across professions and clinical settings, whilst being small enough to maximise within-group efficiency and ensure consensus could be reached. The two 2-hour meetings of the group were facilitated by RP, a clinical pharmacologist and GP with expertise in medicine optimisation. Additional work was conducted remotely, and the group members were reimbursed for their time. Formal ethical permissions were not required.

Meetings and group work employed a standard approach to group decision-making, involving discussion, followed by formation and modification of proposals, and checking agreement of participants. Meetings were structured around a clear agenda, with all group members afforded equal input. Outside of meetings, individual participants’ comments on materials and specific preferences were collated and then shared with the group as a whole prior to further decisions being taken.

To begin, individuals in the group were provided by email with a summary of the systematic review findings, along with brief evidence summaries for each of the clinical themes and core elements of the process and delivery themes (healthcare professional interaction, follow-up, review facilitators) identified by the review, and examples of previous review models. Independent comments were then shared with the group. The initial meeting was used to agree on the main purpose of the review model, to identify potential overarching model structures and to recommend potential review components and supporting material. Following the first meeting, three draft review models were drawn up by the research team (RP, DM, RD), informed by the framework developed from the systematic review, previous sample review models and recommendations from the working group. These draft models were circulated via email to group members for review and comment. A further draft model was drawn up based on resulting feedback and shared along with comments with the group. A second meeting was then held during which the overall model structure was agreed, and further decisions made about the content and supporting materials. The final model was agreed upon by all participants and finalised via email following the third round of refinement and comment.

### Patient and public involvement

Our departmental Patient and Public Involvement in Research advisory group, with specific interests in medicines and prescribing research and comprising patients of a diverse mix of ages, gender and ethnicity, was consulted on the design and purpose of the study. Patients also contributed to the model development as part of the stakeholder working group.

## Results

### Systematic review

**Fig. 1:**
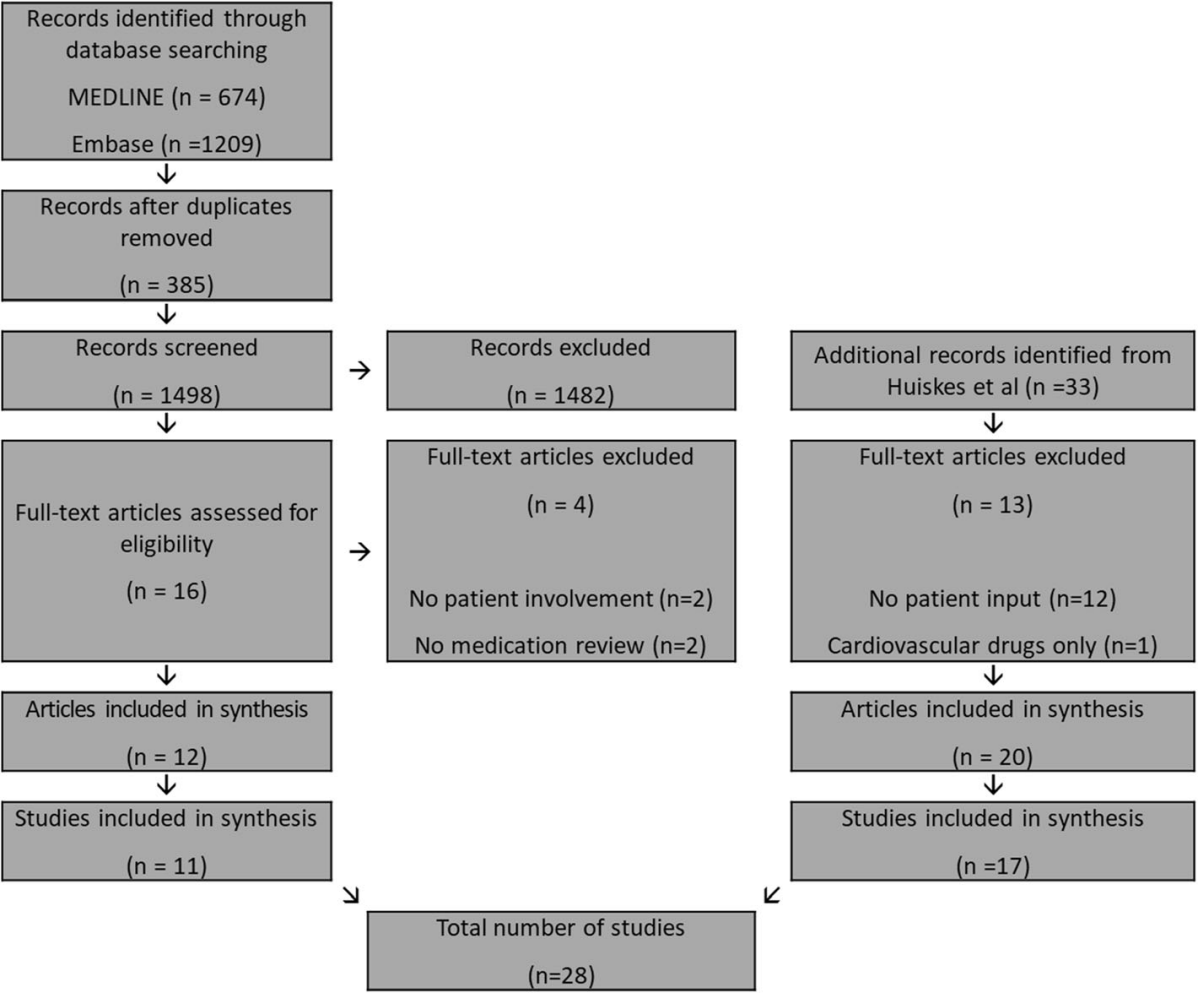
PRISMA flow diagram of the literature search and study selection process

The abstracts of 1498 scientific papers were identified by the updated search, in addition to the original 33 records from Huiskes’ review. Following full-text assessment, a total of 32 articles representing 28 trials met the selection criteria and were included (PRISMA flow diagram, Fig. 1; Additional File 1: Appendices 3 and 4).

The components identified in the different medication review strategies are reported in Table 1. The most frequently used review components were medicines reconciliation (26/28 studies, e.g. determining actual drug use by the patient), an assessment of medicine safety (22 studies, e.g. assessment of side effects, potential for anticholinergic drug effects, contraindications and/or drug-drug interactions), assessment of suboptimal treatment (19 studies, e.g. presence of a valid clinical indication, unnecessary medicines use or undertreatment) and evaluation of patient-orientated issues (18 studies, e.g. patient knowledge and understanding of medicines, patient values and preferences and practical issues pertaining to taking medicines). Less frequently used review components were assessment of medication adherence (14 studies), over-the-counter therapy (13 studies), drug monitoring (10 studies, e.g. drug levels and other biomarkers), one or more drug appropriateness tools (9 studies, e.g. STOPP/START criteria) and drug costs (8 studies).

**Table 1:**
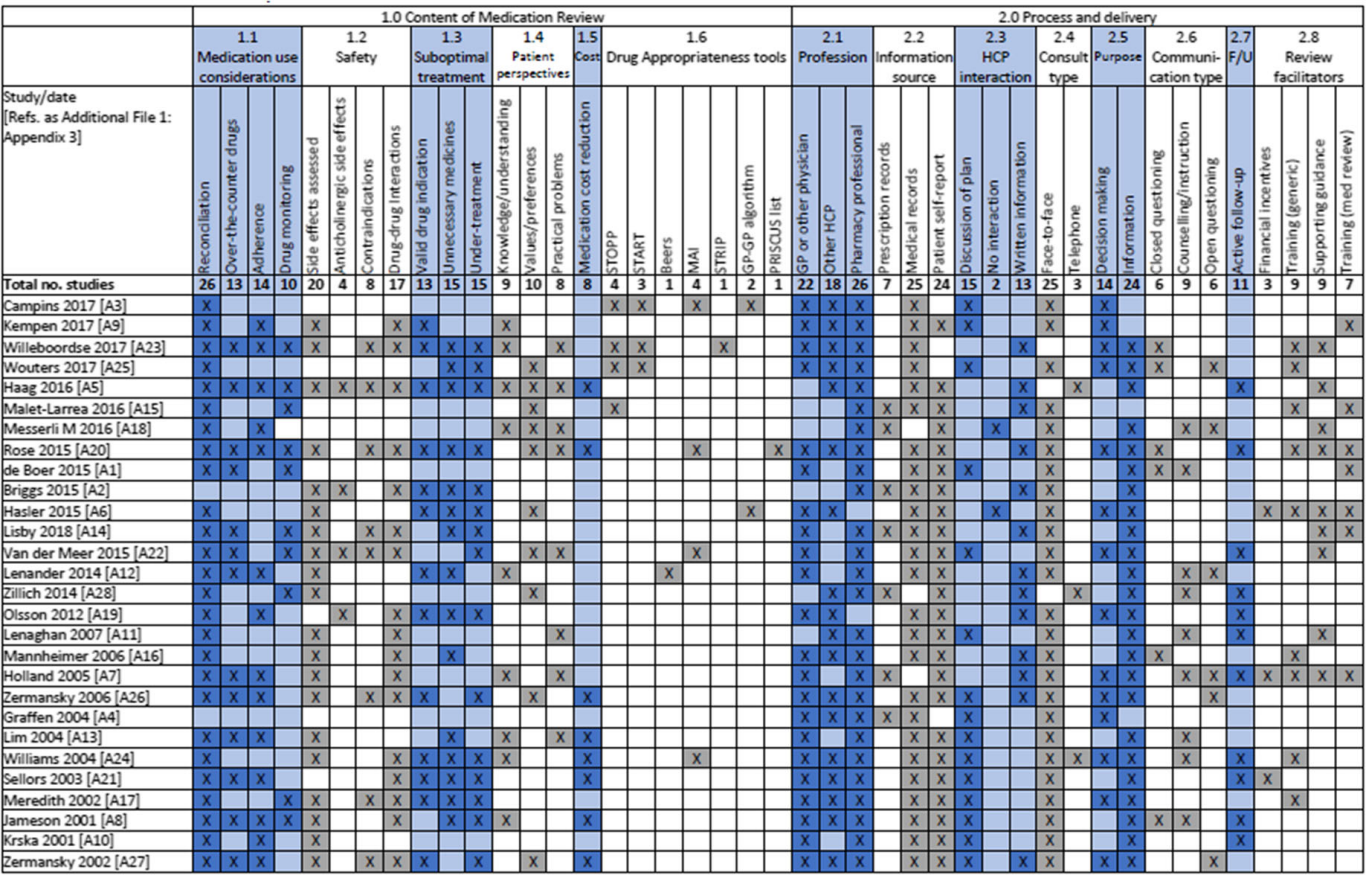
Medication review components identified in different studies

The vast majority of reviews (25/28) involved a face-to-face consultation with the patient, although the exact nature of communication was often not reported (Table 1). Most medication reviews involved input from more than one healthcare profession (25/28) and all but two included pharmacist involvement. Where review decisions were not being actioned by the reviewer themselves, the treatment plan was discussed with another healthcare professional in 15 studies, and written information was provided in 13 studies. Active follow-up formed part of the review process in 11 studies. A number of potential review facilitators were identified, including supplying providers with financial incentives, training and supporting resources.

The outcomes assessed in each study are reported in Table 2. Overall, 15 studies investigated the impact of the medication review intervention on unplanned hospital admissions, 13 examined the total number of medicines prescribed and 11 assessed the outcome of generic drug-related problems. Fifteen studies examined an outcome—quality of life—which forms part of a proposed core outcome set for polypharmacy medication review [21]. Few studies assessed the impact of the medication review strategy on cognitive function (3), number of falls (2), mortality (1) or medication efficacy (1); further analyses of these outcomes were therefore considered inappropriate. Two of 11 studies demonstrated statistically significant improvements in generic drug-related problems, 3 of 9 showed improvements in adherence, 7 of 13 studies reported significant reductions in the number of medicines and 1 of 15 studies showed an improvement in quality of life. A further study showed an increase in unplanned hospital admissions in the intervention group. Due to heterogeneity in intervention design and outcome measures, it was not possible to undertake a meta-analysis or meta-regression of outcomes on review components.

**Table 2:** Categories of outcomes measured in the included studies

Category of outcome	Outcome	No. of studies reporting outcome	No. of studies showing improvement in outcome	References to studies showing improvement (Additional file **1**: Appendix 3)
Service use	Unplanned hospital admissions	15	0^b^	A7^b^
Patient experience	Quality of life^a^	15	1	A12
	Number of medications	13	7	A3, A8, A11, A12, A24, A25, A27
	Medication adherence	9	3	A3, A10, A13
Safety	Drug-related problems	11	4	A10, A17, A22, A23
	Cognitive function	4	1	A22
	Number of falls	4	1	A26
Mortality	Mortality	8	0	–
Cost	Medication cost reduction	6	3	A8, A24, A27
Efficacy	Efficacy	2	1	A25

^a^Part of the core outcome set for polypharmacy medication review (Beuscart et al. [21])

^b^Single study showed an increase in admissions

**Fig. 2:**
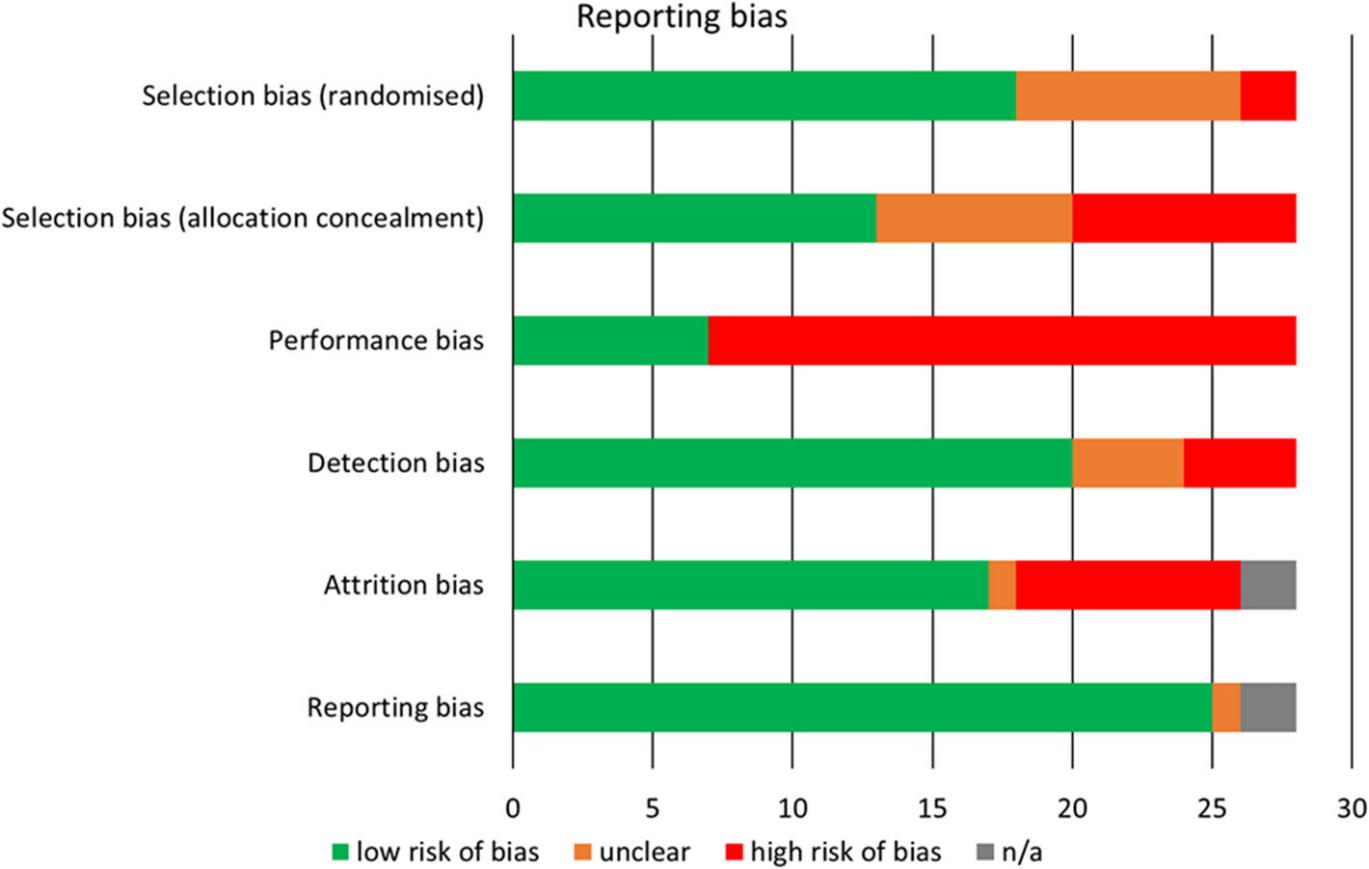
Overall risk of bias

The summary results of the risk of bias assessment are reported in Fig. 2 (individual study risk of bias is reported in Additional File 1: Appendix 5). Overall, included studies had a moderate risk of bias. There was a lack of detail provided by some studies to fully assess bias, particularly methods of randomisation and allocation concealment. It was not possible to blind participants in this type of intervention, and this question was therefore rated as high risk in most cases, although some studies did blind outcome assessors thereby reducing detection bias. Two studies were only available as protocol papers; review components and intended outcomes were extracted but not results.

### Model development

**Fig. 3:**
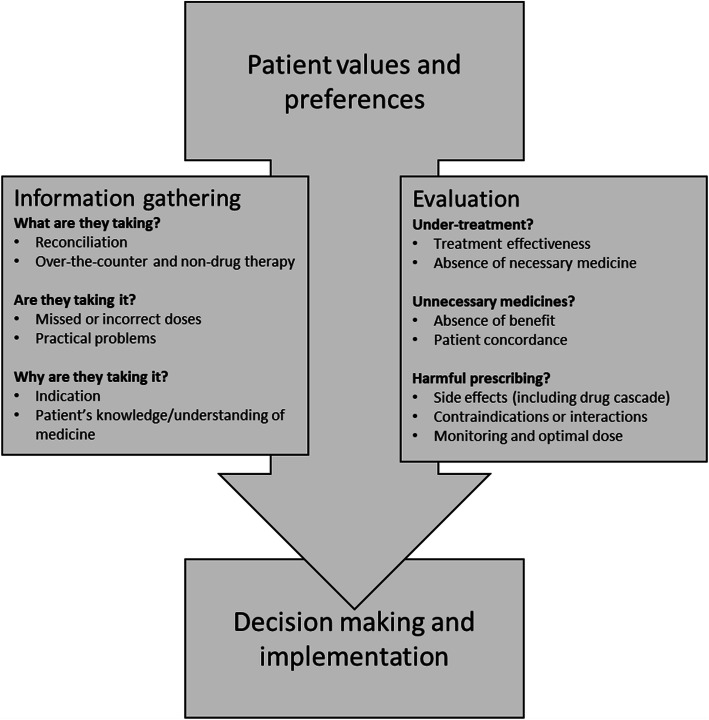
Bristol Medication Review Model

The final model is presented in Fig. 3; the final version with accompanying supporting materials is provided in Additional File 1: Appendix 6.

At the initial meeting, the working group agreed that the resulting structured medication review model should be simple and pragmatic and one that can be used across professions and settings, applied in a flexible way, and include relatively “high-level” detail only.

The group expressed a number of key views about the overarching review model structure, based on the examples presented to it prior to the first meeting, and these informed the subsequent draft models developed by the research team. Firstly, the defined start (patient values and preferences) and end (decision making and implementation) was considered to be more in-keeping with traditional consultation structure and patient expectation, and thus aiding implementation in clinical practice. Secondly, the group felt that the parallel information gathering and evaluation stages facilitated a more holistic approach, making it easier to capture the broader patient story, with the former stage focused more on patient problems and the latter stage on medications. Thirdly, the model was considered by the group to emphasise the importance of patient-centredness.

The first meeting also provided an opportunity to agree on the review components. Whilst acknowledging that all the medication review components identified from the literature were relevant, the working group took a decision to exclude medication cost reduction, on the basis it was less relevant to patient-facing clinical interaction. In addition, the use of potentially inappropriate prescribing tools (e.g. STOPP/START, Beers) was excluded on the basis that these are already captured by other review components. The group also felt that the use of non-pharmacological therapies, and the concept of drug cascade, should be explicitly mentioned by the model.

The second meeting focused on refining the review model and agreeing on the supporting materials. The working group advocated the use of slightly more “technical” terms in the model to aid brevity, given the clinical audience. However, to improve understanding, there was strong backing for additional inclusion of a list of definitions for the terminology used, in turn supported by example open questions suitable for use in clinical practice. Although there was an agreement that the use of the review model should remain flexible and not be limited to a single approach, the group did make several recommendations for implementation. These included advocating the use of pre-review preparation, a preference for in-person face-to-face interaction, use of various review facilitators (e.g. training, dedicated time, financial incentives) and taking care to avoid clinical informatics support (e.g. decision support tools or computerised data-recording templates) or review monitoring processes unduly impairing the clinical process.

## Discussion

It has been possible to use existing studies to develop a new, patient-centred model of review that is potentially suitable for clinical practice which incorporates established components of medication review. There is heterogeneity in the design of various models of medication review that have been studied in clinical trials, and despite patient-centredness being considered core to many medication models, patient perspectives are not captured by around a third of models. The studies identified are not of high quality, do not report the content and structure of interventions in detail and are inconsistent in the outcome measures they examine. Nevertheless, a number of consistent components exist which are appropriate for use in practice, with recurring themes including safety, suboptimal treatment and broader medication use considerations such as adherence. The current study provides some evidence that medication reviews may reduce total numbers of medications and cost and more limited evidence for improvements in adherence and drug-related problems. However, there is no clear evidence that particular elements of these are associated with improvements in specific clinical outcomes. Nonetheless, it has been possible to draw on the existing literature to inform a new review model which is more comprehensive than other published models, has the potential to reduce heterogeneity in existing approaches [16] and may help address barriers to implementation such as complexity and applicability [22].

### Strengths and limitations

The use of formal systematic review methodology has helped ensure the model is comprehensive in terms of its content. The involvement of a range of clinicians together with patients has helped improve acceptability and generalisability. However, there are limitations. In order to isolate the effect of medication review, we restricted studies to those of medication review alone by excluding more complex interventions, and this may have resulted in the omission of important studies. We only extended our search to 2018, and this may again have omitted relevant trials, although we do not believe more recent literature is likely to significantly alter our analytical framework or the resulting model development. We were also unable to use quantitative methods to synthesise our findings as originally proposed in our protocol, due to heterogeneity in the nature of the interventions and in the outcomes reported. This makes it difficult to determine which elements of the review model are most likely to be effective in practice. In addition, our initial intention had been to use the information on the behavioural theory basis of the various interventions to inform the model design, but we were unable to extract this due to insufficient published detail. The stakeholder working group was also relatively small but reflects a trade-off between ensuring representativeness and avoiding difficulties of achieving consensus in a larger group. Finally, further work is required to test acceptability, feasibility and effectiveness in practice.

### Comparison with literature and implications for practice

Others have observed substantial variations in the content of medication reviews [17], and this is reflected in the variability of intervention design we observed in our own study. Our model sought to capture most of the key components identified in our review of the literature, and these correspond with, and indeed are arguably more comprehensive than, those set out by recommendations made in various national and international guidance and standards documents [7, 9, 23, 24]. Although medication costs are considered in some review models, our decision to exclude this is consistent with others’ views that this is less relevant from a clinical and patient perspective [25].

The design of the model emphasises the importance of patient-centredness. This is considered central to medicine optimisation [6] and can improve patient satisfaction and adherence [26]. The structure is also consistent with familiar traditional consultation frameworks such as the Calgary-Cambridge approach [27], which may facilitate implementation in practice. At the same time, the design is flexible enough to allow for the contrasting approaches of doctors and pharmacists, the latter perceived as providing more medication-focused as opposed to person-focused care [28].

It is not possible to say whether the proposed model is effective at optimising the use of medicines in practice, and further evaluation is required. Nevertheless, the model and accompanying example questions and supporting information are consistent with relevant behaviour change theory, addressing psychological capability and both reflective and automatic motivations of the clinician to engage with the medication review process, by providing an easy-to-use resource which supports the development of knowledge and skills, as well as facilitating the planning and delivery of the review process [29]. Flexibility in design is also likely to aid implementation, given it has been argued elsewhere that restrictive guidance may not be well suited to the complexity of clinical practice [30]. There is also evidence to support the recommendations for implementation such as financial incentivisation [31] and provision of relevant training [32]. Several potential barriers to the implementation of guidance have additionally been identified by previous research, including lack of evidence, implausibility, complexity, poor layout, lack of applicability and generalizability [12]. By addressing these issues, we can have greater confidence in the acceptability of the proposed model.

## Conclusion

The Bristol Medication Review Model has been developed by integrating common themes identified in existing studies with a patient-centred approach. The model addresses deficits in existing approaches, emphasising the importance of capturing patient values and preferences. The framework enables reviews to be conducted in a consistent manner, potentially reducing variation in standards of existing reviews. The model is designed to offer flexibility of implementation, with the potential for use by all healthcare professionals with relevant clinical experience across healthcare settings. Evaluation of the acceptability, feasibility and effectiveness of the model will be the subject of future study.

## Supplementary Information

**Additional file 1:.** Appendix 1. Search strategy and full electronic search. Appendix 2. Medication review framework and definitions. Appendix 3. Reference list of included articles, grouped by study intervention. Appendix 4. Excluded articles and reasons for exclusion. Appendix 5. Risk of bias within each study. Appendix 6. Bristol Medication Review Model – practice document.

## Data Availability

Data from the systematic review can be shared on request.
