# Supplementary material for: Development of a model of medication review for use in clinical practice: Bristol medication review model
Source: BMC Med. 2021 Nov 10;19:262. doi: 10.1186/s12916-021-02136-9 (PMC8579564; doi:10.1186/s12916-021-02136-9)
Supplement: Supplementary file 1 — Additional file 1:. Appendix 1. Search strategy and full electronic search. Appendix 2. Medication review framework and definitions. Appendix 3. Reference list of included articles, grouped by study intervention. Appendix 4. Excluded articles and reasons for exclusion. Appendix 5. Risk of bias within each study. Appendix 6. Bristol Medication Review Model – practice document. [file 12916_2021_2136_MOESM1_ESM.docx]

**Development of a model of medication review for use in clinical practice: Bristol Medication Review Model**

**Additional File 1**

**Appendix 1: Search strategy and full electronic search**

Searches of MEDLINE and Embase databases were performed using the following criteria

1.Drug Utilization review/

2.Pharmaceutical services/

3.((pharmacist* or drug or drugs or medication* or medicine* or pharmaceutic* or pharmacology or pharmacologist* or pharmacotherapeutic* or pharmacotherapy or pharmacy or pharmacies or polypharmacy or prescription* or prescribing) adj2 ( intervention or interventions or review or reviews or program or programs or programme or programmes or assessment* or care or counselling or consult* or management or evaluation or evaluations))

4. 1 or 2 or 3

AND

RCT filter

AND

(2015* or 2016* or 2017* or 2018*).ed,ep,dc

**Appendix 2: Medication review framework and definitions**

| **Review component** | | **Details** |
| --- | --- | --- |
| **1.0 Content of medication review** | |  |
| **1.1 Pharmacotherapeutic medication use considerations** | | What specific medication use related factors are considered? |
|  | Actual drug use by the patient established | Medicines reconciliation, with the patient being asked what drugs they are actually taking |
|  | Including over the counter medicines | Use of over-the-counter medicines is considered |
|  | Adherence with drugs assessed | Any type of assessment of adherence - patient reported, refills collected etc |
|  | Drug monitoring needs assessed | Therapeutic monitoring or disease monitoring via laboratory or non-laboratory investigation (to include assessment of symptoms and tests of blood pressure, INR, kidney function/ liver function) |
| 1**.2 Safety** | |  |
|  | Assessment of side effects | Identification of any harms from medicines. Assessment of specific drug related problems such as adverse drug reactions, side effects, drug-drug interactions, and therapeutic duplication |
|  | Potential for anticholinergic side effects from drug |  |
|  | Contraindications considered |  |
|  | Drug-drug Interactions |  |
| **1.3 Suboptimal treatment** | |  |
|  | Valid indication for medicines identified | For each drug used the reason for it being prescribed is reviewed to assess continued need |
|  | Unnecessary medicines | Checking for any evidence of over treatment including duplication of therapy, inappropriate duration of therapy, wrong dosage in terms of dosage being too high according to renal function or age, or medication overuse i.e. patient is taking too much |
|  | Under treatment identified | Checking for any evidence of undertreatment to include; indication with no prescription/treatment, unaddressed medical problems, undertreatment of a condition due to an ineffective drug (i.e insufficient drug dosage according to guidelines or disease state control as indicated by uncontrolled signs and symptoms) or due to wrong dosage of a drug particularly an inappropriate dosing interval (time interval between doses is too long) |
| **1.4 Patient perspectives** | |  |
|  | Patient knowledge/understanding examined | Patient knowledge of what the drug is for and how it should be taken is assessed |
|  | Patient values and preferences considered | Patients are offered the opportunity to express any values or preferences, concerns and questions about their medicines |
|  | Practical problems with medicines assessed | Patient specific problems with use or storage of their medicines, practical/dexterity problems, opening bottles or packets |
| 1**.5 Drug cost** | |  |
|  | Medication cost reduction | Lower cost alternative medicines are specifically sought as part of the review process. |
| **1.6 Drug Appropriateness tools** | | Are drug appropriateness tools used to support the medication review process? If so, which tools? |
|  | STOPP |  |
|  | START |  |
|  | Beers |  |
|  | MAI - Medication appropriateness index |  |
|  | STRIP * |  |
|  | GP-GP algorithm |  |
|  | PRISCUS list |  |
| **2.0 Process and delivery** | |  |
| **2.1 Profession** | | Which healthcare professionals are involved in the scrutiny of the information gathered, formulation of recommendations/decision-making for the treatment plan and implementation of the plan? |
|  | GP or other physician | Includes a GP, physician, doctor |
|  | Other Health Care Professional (HCP) | Nurse, social worker, psychiatrist. physiotherapist |
|  | Pharmacy professional | Includes clinical pharmacist, community pharmacist, clinical pharmacologist, geriatric pharmacist |
| **2.2 Information source** | | What information sources are used to facilitate the medication review? |
|  | Dispensing or prescribing records |  |
|  | Medical records |  |
|  | Patient self report | Includes information gathering via patient completion of a questionnaire, which may or not be administered face to face (e.g includes written self-report by patient) |
| **2.3 Health Care Professional Interaction** | | Do HCPs interact with one another in the process of developing and implementing a treatment plan for the patient? If so, what type of interaction occurs (discussion, written feedback)? |
|  | Health Care Professionals discussed treatment plan | Discussion occurs between health care professionals involved in the formulation and implementation of the treatment plan |
|  | No interaction, one HCP involved | No interaction between health care professionals takes place, treatment planning involves one health care professional and occurs at the time of the review |
|  | Written information prior to implementation | A written treatment plan (formulated by health care professional /s undertaking scrutiny and/or discussion of information gathered) is provided to another Health Care Professional for implementation. The Health Care Professional receiving the plan will not have been directly involved in prior discussion and formulation of the treatment plan |
| **2.4 Consultation type** | | What type of contact is used to communicate with the patient at any point during the medication review process? |
|  | Face to face |  |
|  | Telephone |  |
| **2.5 Purpose of communication** | | What is the purpose of communication with the patient at any point of the medication review process? |
|  | Decision making and treatment planning |  |
|  | Information gathering or provision |  |
|  | Closed questioning |  |
| **2.6 Communication type** | | What type of communication is used with the patient at any point during the medication review process? |
|  | Closed questioning | To include administration and completion of a face-to-face self-reported questionnaire and a written self-reported questionnaire at any point during the review process |
|  | Counselling/instruction/information provision | Provision of written and verbal information to patients as part of the medication review process to included training, education, advice, instruction |
|  | Open questioning or probing |  |
| **2.7 Follow -up** | | Is active follow-up as part of the medication review process? |
|  | Active follow-up as part of the review process |  |
| **2.8 Review facilitators** | | What are the facilitators of medication review? |
|  | Financial incentives |  |
|  | Training to support medication optimisation | Provision of education materials and/or face to face training and education in topics/areas which support medicine optimisation, but is not specific to the medication review intervention |
|  | Resources for medication review content | Provision of education materials (to include written guidelines and checklists) which is specific to the medication review strategy (but not including face to face delivery of information) with a focus upon the content of the medication review |
|  | Training in medication review content | More than just provision of documents/material and involving face to face delivery of education and/ or reviewer time to engage with education resources that are specific to the medication review intervention |

**Appendix 3:** **Reference list of included articles (n=32), grouped by study intervention (n=28)**

| **Ref** | **Article citation** |
| --- | --- |
| **A1** | de Boer M, Boeker EB, Ramrattan MA, et al for the Surgery and Pharmacy in Liaison (SUREPILL) Study Group. Effect of a ward-based pharmacy team on preventable adverse drug events in surgical patients (SUREPILL study). Br J Surg. 2015;102:1204-12 |
| **A2** | Briggs S, Pearce R, Dilworth S, Higgins I, Hullick C, Attia J. Clinical pharmacist review: a randomised controlled trial. Emerg Med Australas. 2015;27:419-26 |
| **A3** | Campins L, Serra-Prat M, Gózalo I, López D, Palomera E, Agustí C, Cabré M; REMEI Group. Randomized controlled trial of an intervention to improve drug appropriateness in community-dwelling polymedicated elderly people. Fam Pract. 2017;34:36-42 |
| **A4** | Graffen M, Kennedy D, Simpson M. Quality use of medicines in the rural ambulant elderly: a pilot study. Rural Remote Health. 2004;4:184 |
| **A5** | Haag JD, Davis AZ, Hoel RW, Armon JJ, Odell LJ, Dierkhising RA, Takahashi PY. Impact of Pharmacist-Provided Medication Therapy Management on Healthcare Quality and Utilization in Recently Discharged Elderly Patients. Am Health Drug Benefits. 2016;9:259-68 |
| **A6** | Hasler S, Senn O, Rosemann T, Neuner-Jehle S. Effect of a patient-centered drug review on polypharmacy in primary care patients: study protocol for a cluster-randomized controlled trial. Trials. 2015;16:380 |
| **A7** | Holland R, Lenaghan E, Harvey I, Smith R, Shepstone L, Lipp A, Christou M, Evans D, Hand C. Does home based medication review keep older people out of hospital? The HOMER randomised controlled trial. BMJ. 2005;330:293 |
|  | Pacini M, Smith RD, Wilson EC, Holland R. Home-based medication review in older people: is it cost effective? Pharmacoeconomics. 2007;25:171-80 |
| **A8** | Jameson JP, VanNoord GR. Pharmacotherapy consultation on polypharmacy patients in ambulatory care. Ann Pharmacother. 2001;35:835-40 |
|  | Jameson J, VanNoord G, Vanderwoud K. The impact of a pharmacotherapy consultation on the cost and outcome of medical therapy. J Fam Pract. 1995;41:469-72 |
| **A9** | Kempen TGH, Bertilsson M, Lindner KJ, Sulku J, Nielsen EI, Högberg A, Vikerfors T, Melhus H, Gillespie U. Medication Reviews Bridging Healthcare (MedBridge): Study protocol for a pragmatic cluster-randomised crossover trial. Contemp Clin Trials. 2017;61:126-132 |
| **A10** | Krska J, Cromarty JA, Arris F, Jamieson D, Hansford D, Duffus PR, Downie G, Seymour DG. Pharmacist-led medication review in patients over 65: a randomized, controlled trial in primary care. Age Ageing. 2001;30:205-11 |
| **A11** | Lenaghan E, Holland R, Brooks A. Home-based medication review in a high risk elderly population in primary care--the POLYMED randomised controlled trial. Age Ageing. 2007;36:292-7 |
| **A12** | Lenander C, Elfsson B, Danielsson B, Midlöv P, Hasselström J. Effects of a pharmacist-led structured medication review in primary care on drug-related problems and hospital admission rates: a randomized controlled trial. Scand J Prim Health Care. 2014;32:180-6 |
| **A13** | Lim WS, Low HN, Chan SP, Chen HN, Ding YY, Tan TL. Impact of a pharmacist consult clinic on a hospital-based geriatric outpatient clinic in Singapore. Ann Acad Med Singap. 2004;33:220-7 |
| **A14** | Lisby M, Bonnerup DK, Brock B, Gregersen PA, Jensen J, Larsen ML, Rungby J, Sonne J, Mainz J, Nielsen LP. Medication Review and Patient Outcomes in an Orthopedic Department: A Randomized Controlled Study. J Patient Saf. 2018;14:74-81 |
| **A15** | Malet-Larrea A, Goyenechea E, García-Cárdenas V, Calvo B, Arteche JM, Aranegui P, Zubeldia JJ, Gastelurrutia MA, Martínez-Martínez F, Benrimoj SI. The impact of a medication review with follow-up service on hospital admissions in aged polypharmacy patients. Br J Clin Pharmacol. 2016;82:831-8 |
|  | Jódar-Sánchez F, Malet-Larrea A, Martín JJ, García-Mochón L, López Del Amo MP, Martínez-Martínez F, Gastelurrutia-Garralda MA, García-Cárdenas V, Sabater-Hernández D, Sáez-Benito L, Benrimoj SI. Cost-utility analysis of a medication review with follow-up service for older adults with polypharmacy in community pharmacies in Spain: the conSIGUE program. Pharmacoeconomics. 2015;33:599-610 |
| **A16** | Mannheimer B, Ulfvarson J, Eklöf S, Bergqvist M, Andersén-Karlsson E, Pettersson H, von Bahr C. Drug-related problems and pharmacotherapeutic advisory intervention at a medicine clinic. Eur J Clin Pharmacol. 2006;62:1075-81 |
| **A17** | Meredith S, Feldman P, Frey D, Giammarco L, Hall K, Arnold K, Brown NJ, Ray WA. Improving medication use in newly admitted home healthcare patients: a randomized controlled trial. J Am Geriatr Soc. 2002;50:1484-91 |
| **A18** | Messerli M, Blozik E, Vriends N, Hersberger KE. Impact of a community pharmacist-led medication review on medicines use in patients on polypharmacy--a prospective randomised controlled trial. BMC Health Serv Res. 2016;16:145 |
| **A19** | Olsson IN, Runnamo R, Engfeldt P. Drug treatment in the elderly: an intervention in primary care to enhance prescription quality and quality of life. Scand J Prim Health Care. 2012;30:3-9 |
| **A20** | Rose O, Schaffert C, Czarnecki K, Mennemann HS, Waltering I, Hamacher S, Felsch M, Herich L, Köberlein J. Effect evaluation of an interprofessional medication therapy management approach for multimorbid patients in primary care: a cluster-randomized controlled trial in community care (WestGem study protocol). BMC Fam Pract. 2015;16:84 |
| **A21** | Sellors J, Kaczorowski J, Sellors C, Dolovich L, Woodward C, Willan A, Goeree R, Cosby R, Trim K, Sebaldt R, Howard M, Hardcastle L, Poston J. A randomized controlled trial of a pharmacist consultation program for family physicians and their elderly patients. CMAJ. 2003;169:17-22 |
|  | Sellors C, Dalby DM, Howard M, Kaczorowski J, Sellors J. A Pharmacist Consultation Service in Community-Based Family Practices: A Randomized, Controlled Trial in Seniors. J Pharmacy Technology 2001;17:264-69 |
| **A22** | van der Meer HG, Wouters H, van Hulten R, Pras N, Taxis K. Decreasing the load? Is a Multidisciplinary Multistep Medication Review in older people an effective intervention to reduce a patient's Drug Burden Index? Protocol of a randomised controlled trial. BMJ Open. 2015;5:e009213 |
| **A23** | Willeboordse F, Schellevis FG, Chau SH, Hugtenburg JG, Elders PJM. The effectiveness of optimised clinical medication reviews for geriatric patients: Opti-Med a cluster randomised controlled trial. Fam Pract. 2017;34:437-445 |
| **A24** | Williams ME, Pulliam CC, Hunter R, Johnson TM, Owens JE, Kincaid J, Porter C, Koch G. The short-term effect of interdisciplinary medication review on function and cost in ambulatory elderly people. J Am Geriatr Soc. 2004;52:93-8 |
| **A25** | Wouters H, Scheper J, Koning H, Brouwer C, Twisk JW, van der Meer H, Boersma F, Zuidema SU, Taxis K. Discontinuing Inappropriate Medication Use in Nursing Home Residents: A Cluster Randomized Controlled Trial. Ann Intern Med. 2017;167:609-617 |
| **A26** | Zermansky AG, Alldred DP, Petty DR, Raynor DK, Freemantle N, Eastaugh J, Bowie P. Clinical medication review by a pharmacist of elderly people living in care homes--randomised controlled trial. Age Ageing. 2006;35:586-91 |
| **A27** | Zermansky AG, Petty DR, Raynor DK, Lowe CJ, Freemantle N, Vail A. Clinical medication review by a pharmacist of patients on repeat prescriptions in general practice: a randomised controlled trial. Health Technol Assess. 2002;6:1-86 |
| **A28** | Zillich AJ, Snyder ME, Frail CK, Lewis JL, Deshotels D, Dunham P, Jaynes HA, Sutherland JM. A randomized, controlled pragmatic trial of telephonic medication therapy management to reduce hospitalization in home health patients. Health Serv Res. 2014;49:1537-54 |

**Appendix 4:** **Excluded articles and reasons for exclusion (n=17)**

| **Article citation** | **Reason for exclusion** |
| --- | --- |
| Bond CM, Fish A, Porteous TH, Reid JP, Scott A, Antonazzo E. A randomised controlled trial of the effects of note-based medication review by community pharmacists on prescribing of cardiovascular drugs in general practice. Int J Pharmacy Practice 2007;15:39-46 | Review of drugs for treatment of cardiovascular disease only |
| Britton ML, Lurvey PL. Impact of medication profile review on prescribing in a general medicine clinic. Am J Hosp Pharm 1991;48:265-270 | No patient involvement |
| Burns A, Furniss L, Cooke J, Lloyd Craig SK, Scobie S. Pharmacist medication review in nursing homes: A cost analysis. Int J Geriatr Psychopharmacol 2000;2:137-141 | No patient involvement |
| Furniss L, Burns A, Craig SK, Scobie S, Cooke J, Faragher B. Effects of a pharmacist's medication review in nursing homes. Randomised controlled trial. Br J Psychiatry. 2000;176:563-7 | No patient involvement |
| Gallagher PF, O'Connor MN, O'Mahony D. Prevention of potentially inappropriate prescribing for elderly patients: a randomized controlled trial using STOPP/START criteria. Clin Pharmacol Ther. 2011;89:845-54 | No patient involvement |
| Heselmans A, van Krieken J, Cootjans S, Nagels K, Filliers D, Dillen K, De Broe S, Ramaekers D. Medication review by a clinical pharmacist at the transfer point from ICU to ward: a randomized controlled trial. J Clin Pharm Ther. 2015;40:578-583 | No patient involvement |
| Hohl CM, Wickham ME, Sobolev B, Perry JJ, Sivilotti ML, Garrison S, Lang E, Brasher P, Doyle-Waters MM, Brar B, Rowe BH, Lexchin J, Holland R. The effect of early in-hospital medication review on health outcomes: a systematic review. Br J Clin Pharmacol. 2015;80:51-61 | No patient involvement |
| Kwint HF, Faber A, Gussekloo J, Bouvy ML. Effects of medication review on drug-related problems in patients using automated drug-dispensing systems: a pragmatic randomized controlled study. Drugs Aging. 2011;28:305-14 | No patient involvement |
| Leguelinel-Blache G, Castelli C, Roux-Marson C, Bouvet S, Andrieu S, Cestac P, Collomp R, Landais P, Loulière B, Mouchoux C, Varin R, Allenet B; MEDREV Working Group, Bedouch P, Kinowski JM. Impact of collaborative pharmaceutical care on in-patients' medication safety: study protocol for a stepped wedge cluster randomized trial (MEDREV study). Trials. 2018;19:19 | No patient involvement |
| Lisby M, Thomsen A, Nielsen LP, Lyhne NM, Breum-Leer C, Fredberg U, Jørgensen H, Brock B. The effect of systematic medication review in elderly patients admitted to an acute ward of internal medicine. Basic Clin Pharmacol Toxicol. 2010;106:422-7 | No patient involvement |
| Meyer TJ, Van Kooten D, Marsh S, Prochazka AV. Reduction of polypharmacy by feedback to clinicians. J Gen Intern Med. 1991;6:133-6 | No patient involvement |
| Michalek C, Wehling M, Schlitzer J, Frohnhofen H. Effects of "Fit fOR The Aged" (FORTA) on pharmacotherapy and clinical endpoints--a pilot randomized controlled study. Eur J Clin Pharmacol. 2014;70:1261-7 | No patient involvement |
| Miguel A, Hall A, Liu W, Garrett J, Ballew A, Yang TH, Segal R. Improving Comprehensive Medication Review Acceptance by Using a Standardized Recruitment Script: A Randomized Control Trial. J Manag Care Spec Pharm. 2017;23:13-21. | Not an evaluation of a CMR, but of a marketing phone-call for insurance company that offers CMR |
| Milos V, Rekman E, Bondesson Å, Eriksson T, Jakobsson U, Westerlund T, Midlöv P. Improving the quality of pharmacotherapy in elderly primary care patients through medication reviews: a randomised controlled study. Drugs Aging. 2013;30:235-46 | No patient involvement |
| O'Connor MN, O'Sullivan D, Gallagher PF, Eustace J, Byrne S, O'Mahony D. Prevention of Hospital-Acquired Adverse Drug Reactions in Older People Using Screening Tool of Older Persons' Prescriptions and Screening Tool to Alert to Right Treatment Criteria: A Cluster Randomized Controlled Trial. J Am Geriatr Soc. 2016;64:1558-66 | No patient involvement |
| Pit SW, Byles JE, Henry DA, Holt L, Hansen V, Bowman DA. A Quality Use of Medicines program for general practitioners and older people: a cluster randomised controlled trial. Med J Aust. 2007;187:23-30 | Patients involvement wasn’t explicit in the intervention. Intervention actually educating clinicians about how to perform medication reviews, then medication review itself, only 3 drugs classes reviewed |
| Pope G, Wall N, Peters CM, O'Connor M, Saunders J, O'Sullivan C, Donnelly TM, Walsh T, Jackson S, Lyons D, Clinch D. Specialist medication review does not benefit short-term outcomes and net costs in continuing-care patients. Age Ageing. 2011;40:307-12 | No patient involvement |

**Appendix 5:** **Risk of bias within each study**

|  | **Random sequence generation**  **(selection bias)** | **Allocation concealment**  **(selection bias)** | **Blinding of participants and personnel**  **(performance bias)** | **Blinding of outcome assessment**  **(detection bias)** | **Incomplete outcome data**  **(attrition bias)** | **Selective outcome reporting?**  **(reporting bias)** |
| --- | --- | --- | --- | --- | --- | --- |
| Campins 2017 [A3] |  |  |  |  |  |  |
| Kempen 2017 [A9] |  |  |  |  |  |  |
| Willeboordse 2017 [A23] |  |  |  |  |  |  |
| Wouters 2017 [A25] |  |  |  |  |  |  |
| Haag 2016 [A5] |  |  |  |  |  |  |
| Malet-Larrea 2016 [A15] |  |  |  |  |  |  |
| Messerli M 2016 [A18] |  |  |  |  |  |  |
| Rose 2015 [A20] |  |  |  |  |  |  |
| de Boer 2015 [A1] |  |  |  |  |  |  |
| Briggs 2015 [A2] |  |  |  |  |  |  |
| Hasler 2015 [A6] |  |  |  |  |  |  |
| Lisby 2018 [A14] |  |  |  |  |  |  |
| Van der Meer 2015 [A22] |  |  |  |  |  |  |
| Lenander 2014 [A12] |  |  |  |  |  |  |
| Zillich 2014 [A28] |  |  |  |  |  |  |
| Olsson 2012 [A19] |  |  |  |  |  |  |
| Lenaghan 2007 [A11] |  |  |  |  |  |  |
| Mannheimer 2006 [A16] |  |  |  |  |  |  |
| Holland 2005 [A7] |  |  |  |  |  |  |
| Zermansky 2006 [A26] |  |  |  |  |  |  |
| Graffen 2004 [A4] |  |  |  |  |  |  |
| Lim 2004 [A13] |  |  |  |  |  |  |
| Williams 2004 [A24] |  |  |  |  |  |  |
| Sellors 2003 [A21] |  |  |  |  |  |  |
| Meredith 2002 [A17] |  |  |  |  |  |  |
| Jameson 2001 [A8] |  |  |  |  |  |  |
| Krska 2001 [A10] |  |  |  |  |  |  |
| Zermansky 2002 [A27] |  |  |  |  |  |  |

|  | Low risk of bias |
| --- | --- |
|  | Unclear |
|  | High risk of bias |
|  | Protocol paper only, unable to assess |

**Appendix 6:** **Bristol Medication Review Model – practice document**

**Bristol Medication Review Model**

The Bristol Medication Review Model **is for use by all health care professionals** with appropriate clinical experience. It is designed **to facilitate patient-facing medication review**, but may also be used as a framework to underpin interprofessional interaction and medication review in the absence of the patient.

**Purpose of a patient-facing medication review**

The purpose of a review should be to understand and inform the patient’s ideas, concerns and treatment goals, considering both clinical priorities and patient preferences, in order to empower the patient and clinician to make informed-shared decisions around the effective and safe use of their medicines. Reviews should be flexible in terms of process and outcome, and clinical considerations should help to facilitate the review process whilst adhering to a principle of maximal possible autonomy for the patient.

**The Model**


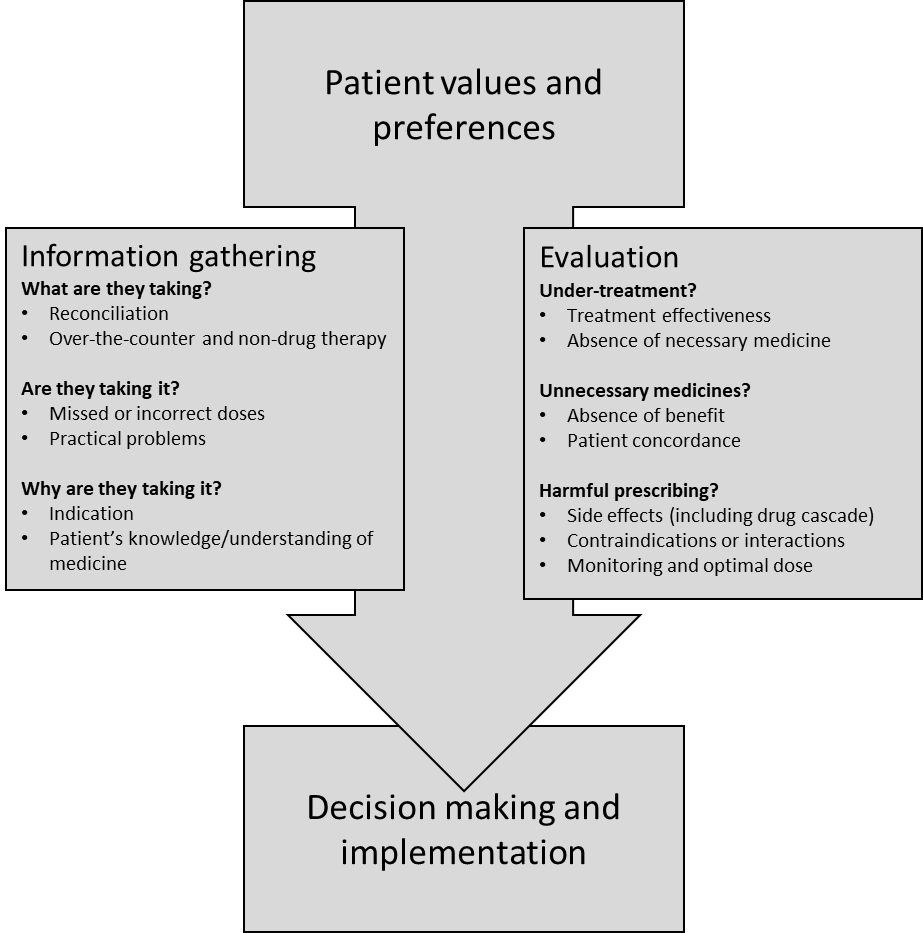


**Recommendations for implementation**

The Bristol Medication Review Model is designed to be **flexible** in terms of how it is implemented, including the healthcare setting in which it is carried out. No specific approach is mandated, although consideration may be given to the following points:

- A “pre-review” process is recommended to ensure adequate preparation for the patient-facing element of the review (see below).
- Information gathering may be improved by including **multiple sources of information**, and ensuring **questions are patient-centred and open** in nature.
- **Face-to-face interaction** is ideal for making patient-centred discussion easier. However, the model also lends itself to video or telephone consultation where this is needed.
- The review process may be facilitated by providing **training for health care professionals**, ensuring **dedicated time** is allotted for carrying out the review, and considering **provision of financial or other incentives** to providers.
- Computerised “templates” may be helpful to record and cue elements of the review, and in particular safety issues. However, they may also limit the flexibility and patient-centredness of the model. The use of templates will therefore depend on individual requirements.
- Measurement and recording of clinical, patient or process outcomes may be of value, and should be adapted according to clinical context and health service need. Any monitoring should be implemented in such a way that it does not actively influence the patient and clinical focus of the review.
- Medication review should be an ongoing process and undertaken on a regular basis appropriate to clinical and patient need. Review reminders may be facilitated using clinical informatics systems. It may be particularly beneficial at points of transition in care (e.g. hospital discharge, admission to residential care settings).

**The pre-review process**

The “pre-review” helps ensure there is adequate preparation for the patient-facing element of any review, and may serve three key purposes:

- **Prepare the patient:** explain the rationale for the upcoming review, and help the patient prepare any questions they might have so their concerns can be addressed fully.
- **Focus the review:** identify patient values and perspectives from the outset, making it clear which components will be most relevant and valuable to spend time on.
- **Gather information:** about medication, problems, priorities. In particular, pharmacological-related aspects of the medication used can be examined, saving time for the patient-facing review.

Use of a pre-review patient questionnaire/leaflet may provide useful background for the discussion. Ideally this should include a space for patients to list specific medications and problems. It should also ask additional open questions (e.g. patient uncertainties, concerns, problems) as per the examples provided for the patient-facing review.

It may be helpful to use a medication table to provide a visual summary to aid discussion of adjustments in the context of patient priorities. For each medication, include clinical indications (or absence of indication) and the purposes of the medication (e.g. disease prevention, symptom management, etc). It may be possible to do this using existing clinical informatics systems.

**Glossary of terms used within the model, and examples of useful corresponding questions**

|  | **Terms and definitions** | **Example questions for patient-facing review** |
| --- | --- | --- |
| **Values and preferences** | Values and preferences are the patient’s general thoughts about taking their medicines. Values are the extent to which a patient considers something to be desirable or undesirable. Patient preferences result from cognition, experience and reflection and exist as the relatively enduring consequences of values. Preferences are always sensitive to context. | - What do you want to get out of your medicines? - What matters most to you in terms of your health and the future? - If you have any concerns about your medicines, what are they? |
| **Information gathering** | ***What are they taking?***  **Reconciliation:** the process of finding out what the person is actually taking by creating the most accurate list possible of all medications being taken (including dose, frequency, route, etc) and comparing the list with all sources of available information (e.g. GP records, hospital records, discharge orders, patient/carer report).  **Over the counter (OTC) medicines:** medicines available without prescription, either with or without pharmacist supervision (Pharmacy Medicines and General Sales List respectively), including multiple products for minor illnesses and certain medicines at restricted dose or pack size that are otherwise provided by prescription.  **Non-drug therapy:** may include conventional measures (e.g. lifestyle measures, physiotherapy, cognitive therapy), alternative treatments (e.g. acupuncture, reflexology) and examples of social prescribing (e.g. arts, volunteering, learning) | - Which medicines are you currently taking, and how? - Which medicines do you take that are not prescribed for you, including herbal remedies or homeopathy? - What treatments are you receiving that are not medicines? |
|  | ***Are they taking it?***  **Missed or incorrect doses:** Patients may miss doses of medicines or take the incorrect dose (including lower, higher or additional doses) either unintentionally (e.g. forgetfulness, misunderstanding) or intentionally (e.g. due to specific beliefs around the benefits and risks of treatment).  **Practical problems:** Difficulties administering medicines due to impaired cognition, difficulties swallowing, vision and manual dexterity, or issues related to renewing or collecting prescriptions. | - Lots of patients forget to take their medicines or cannot take doses of their medicines. How does this affect you? - Do you sometimes take a different dose to the one prescribed? Why? - Do you have problems taking your medicines regularly? - Some people have difficulty swallowing medicines or ordering or collecting medicines. Does this affect you? |
|  | ***Why are they taking it?***  **Indication for the drug:** the presence of a valid, current clinical condition, situation, or reason to justify the use of the medication.  **Patient knowledge and understanding of medication:** This may be influenced by several factors, including education, beliefs and culture. It covers aspects such as the purpose of the medicine, dosage, scheduling, side effect risks, interactions, associated lifestyle changes, and monitoring. | - Are there any medications you are unsure about why you are taking them or how to use them? |

|  | **Terms and definitions** | **Example questions for patient-facing review** |
| --- | --- | --- |
| **Evaluation** | ***Under-treatment?***  Under-treatment is the omission of drug therapy that is indicated for the treatment or prevention of a disease or condition.  **Effectiveness:** achieving the expected pharmacological therapeutic effect, as determined by a relevant patient or clinical outcome. | - Are there any medicines you would like to talk about increasing or starting? - Do you feel that your symptoms are well controlled? |
|  | ***Unnecessary medicines***  Those medicines prescribed despite there being no clear clinical benefits, for example due to a lack of clinical improvement, the patient being unlikely to get benefit (e.g. due to frailty or limited life expectancy), or lack of a robust underlying evidence base.  **Concordance:** Agreement between healthcare professional and patient regarding whether and how medicines will be taken. Medicines may be considered unnecessary by patients, or at odds with their other priorities, and they do not wish to take it. | - Do you feel that any of your medicines are unnecessary and if so why? - Are there any drugs that you feel no longer help you? |
|  | ***Harmful prescribing***  **Side effects:** unwanted reactions (potentially harmful or unpleasant), resulting from an intervention related to the use of a medicinal product, which predicts hazard from future administration and warrants prevention or specific treatment, or alteration of the dosage regimen, or withdrawal of the product.  **Drug cascade:** the process whereby a further prescription is issued to treat or prevent side effects of an existing drug. This is an important cause of potentially avoidable polypharmacy.  **Contraindications:** a specific clinical situation (e.g. condition, diagnosis, syndrome) in which a medication should not be used because it may be harmful to the person, either due to exacerbation of the condition in question, or due to altered, potentially harmful, drug actions.  **Interactions:** co-prescriptions to be avoided, or which should only be used with caution and appropriate monitoring, due to unwanted and potentially hazardous or unpredictable pharmacokinetic or pharmacodynamic consequences.  **Monitoring:** 1) therapeutic (pharmacokinetic) drug monitoring, where medication levels are measured in the blood (especially drugs with a narrow therapeutic range), or 2) monitoring of clinical parameters to assess clinical effectiveness (e.g. INR testing for warfarin, blood pressure for antihypertensives) or safety (e.g. renal function for ACE inhibitors). Monitoring reduces uncertainty in the risk-benefit profile of medications. Consider the type, frequency and duration of monitoring.  **Optimal dose:** achieves desired clinical effect with minimum likelihood of undesirable symptoms. | - Are any of your medicines causing problems? - Are you experiencing any unwanted or unexpected effects from your medicines? [consider prompting with examples based on serious or common known side effects] - Do you have any symptoms you are worried might be due to your medicines? - Are there any difficulties with the monitoring of your medicines? For example, are you able to easily attend appointments for blood tests? |
